# The upside of stress: a mechanism for the positive motivational role of corticotropin releasing factor

**DOI:** 10.1038/s41386-019-0510-9

**Published:** 2019-09-11

**Authors:** Julia C. Lemos, Veronica A. Alvarez

**Affiliations:** 1 Department of Neuroscience, University of Minnesota, Minneapolis, MN 55455 USA; 2 National Institute on Alcohol Abuse and Alcoholism, Bethesda, MD 20892 USA; 3 National Institute on Drug Abuse, Bethesda, MD 20892 USA; 4 Center on Compulsive Behaviors, Intramural Research Program, NIH, Bethesda, MD 20892 USA

**Keywords:** Cellular neuroscience, Motivation

Intuitively, we know that salient environmental stimuli, even when stressful, can trigger an internal state of urgency that focuses our attention, motivates us to work harder, encourages us to explore new spaces or people, and helps us achieve specific goals. Despite its known benefits, the neural mechanisms that underlie these positive motivational qualities of acute stress remain poorly understood. In the past 10 years, evidence has emerged pointing to a new role for the neuropeptide corticotropin-releasing factor (CRF). CRF is a well-studied stress-associated neuropeptide. A rich literature exists demonstrating neuronal substrates of CRF-evoked energy mobilization, fear, and anxiety. However, when acting in the nucleus accumbens (NAc), CRF has been shown to promote exploratory behaviors and invigoration for reward [1, 2]. Yet, the cellular mechanism(s) mediating these positive motivational actions of CRF in the NAc were not known.

In a study published two months ago, we showed that CRF type 1 receptors (CRF-R1) are ubiquitously expressed on cholinergic interneurons within the NAc of adult male mice [3]. We found that CRF produces a robust increase in action potential firing in cholinergic interneurons via CRF-R1 activation and cAMP dependent mechanisms [3]. Cholinergic interneurons form dense axonal ramifications, and therefore, through acetylcholine modulation, can act as master regulators of accumbal output. Previous work had shown that selective ablation of cholinergic interneurons disrupts locomotion and enhanced dopamine transmission triggered by an acute stressor, indicating this cell population plays a critical role in adaptive stress processing [4]. Moreover, work from the Greengard laboratory and others has shown that suppression of cholinergic interneuron firing through a variety of cell-type specific transgenic or chemogenetic manipulations produces behaviors consistent with a depression-like state in mice [5] that is accompanied by a reduction in reward-evoked dopamine release [6]. Thus, we reasoned that the CRF mediated increase of cholinergic interneuron firing may facilitate pathways that drive the opposite behaviors: active coping and hedonic behaviors such as reward consumption. This notion is supported by our original findings that CRF both potentiates dopamine and facilitates appetitive behaviors when acting in the NAc. In this more recent report, we further show that this effect is, in part, due to activation of muscarinic type 5 receptors (M5) on dopamine neuron projections [3]. We propose that CRF’s potentiation of cholinergic and dopaminergic transmission in the NAc is an underlying mechanism for the positive motivational qualities of acute stressors.

We further speculate that vulnerabilities to neuropsychiatric diseases such as anxiety, depression, and addiction may develop due to a diminution of the positive qualities of stress, not only an exacerbation of the negative qualities of stress. Studies in human subjects demonstrate disfunction in NAc activity in patients with depression compared to healthy controls in response to positive and negative stimuli using fMRI [7, 8]. Moreover, in mice, Gαs-DREADD activation in accumbal cholinergic interneurons was able to rescue depression-like phenotypes induced by chronic stress [5]. Thus, we remain hopeful that as we expand our understanding of the neuronal substrates that underlie the positive motivational qualities of stress and stress-associated neuropeptides like CRF, we may get closer to understanding the etiology of these diseases and improve treatment.

## Funding and disclosure

This study was funded by the Intramural Programs of NIAAA, NINDS (ZIA-AA000421) to VAA, K99/R00 Pathway to Independence award (MH109627) to JCL and 2017 Innovation Award from NIH-DDIR to VAA.
